# Clinical and epidemiological investigation of a child with asymptomatic COVID-19 infection following reoccurrence

**DOI:** 10.1007/s13755-022-00188-6

**Published:** 2022-08-24

**Authors:** Qiu‑Yu Lin, Guo‑Tian Lin, Fan Zhang, Xia-Yu Xiang, Yue‑Hua Zhang, Jia‑Chong Wang, Yu‑Ming Jin, Yuan-ping Hai, Tao-Wu, Zhi‑Yue Lv, Wei Xiang

**Affiliations:** 1 Hainan Women and Children’s Medical Center, Children’s Hospital of Fudan University at Hainan, Children’s Hospital of Hainan Medical University, Haikou, 570311 China; 2 Laboratory of Tropical Environment and Health, School of Public Health, Hainan Medical University, No. 3, Chengxi Xue yuan Road, Longhua District, Haikou, 571199 China; 3 Peng Cheng Laboratory, Shenzhen, Guangdong China; 4 People’s Hospital, Central South University Xiangya School of Medicine Affiliated Haikou Hospital, Haikou, 570203 China; 5 Hainan Provincial Center for Disease Control and Prevention, Haikou, 570203 China; 6 Hainan Affiliated Hospital of Hainan Medical University, Haikou, 570311 China; 7 Key Laboratory of Tropical Translational Medicine of Ministry of Education, Hainan Medical University, Haikou, Hainan China; 8 NHC Key Laboratory of Control of Tropical Diseases (Hainan Medical University), Haikou, 571199 Hainan China

**Keywords:** COVID-19, Children, Reactivation, Recurrent infection

## Abstract

**Objective:**

To investigate the case of a child infected with coronavirus disease 2019 (COVID-19) who had subsequent viral reactivation.

**Methods:**

We retrospectively analyzed the clinical manifestations, epidemiological data, laboratory and imaging examinations, treatment, and follow-up of the child. And then, we searched related literature using PubMed.

**Results:**

The 9-year-old boy was exposed to COVID-19 in Malawi and tested positive for NAT in Haikou, China. He was asymptomatic and admitted to our hospital. After six negative NATs, he was discharged from the hospital and quarantined in a hotel. His infection was reactivated again after 22 days (interval between first and last positive NATs). The cycle threshold (Ct) values of positive tests were 25 and 31, and the gene sequencing viral loads were very low. The viral strain Kenya/P2601/2020, a variant of the hCoV-19/Wuhan/IVDC-HB-01/2019 genome (GISAID accession IL: EPI_ISL_402119), was found when polymerase chain reaction enrichment was used to sequence the virus. However, people around him tested negative for COVID-19.

**Conclusion:**

First, we confirmed the reactivation of COVID-19 in a child. The risk of recurrent infection with SARS-CoV-2 was low, and the policy of strictly isolating patients carrying long-term viral ribonucleic acid should be reconsidered. The interval positivity was most likely due to incorrect sampling and/or testing methods. SGS and aB testing are recommended for children with viral reactivation. Second, SARS-CoV-2 viral reactivation cannot be ruled out. The possible mechanisms, such as prolonged infection and viral latent reactivation, need further investigation.

## Introduction

The coronavirus disease 2019 (COVID-19) virus, severe acute respiratory syndrome coronavirus 2 (SARS-CoV-2), and its clinical manifestations vary from person to person due to different viral subtypes and their variants. Nucleic acid tests (NATs) and antibody (aB) detection are the most common screening methods. A positive result is defined as the prolonged presence of viral ribonucleic acid (RNA), reoccurrence of disease positivity, or disease reactivation. “Reactivation” refers to improvement after treatment in patients diagnosed with COVID-19, asymptomatic infection, infection without fever or respiratory symptoms, infection with significant improvement in acute exudative lesions on pulmonary imaging, at least two consecutive NATs that are negative for respiratory specimens at the end of the isolation period (at least 24 h between samples), reoccurrence of positivity for respiratory secretions (mainly pharyngeal swabs), and/or positive results on other viral NATs (such as anal swabs) during recovery. Re-positive patients have brought a new challenge to the global battle against COVID-19, as discharged patients with viral activity are a potential source of transmission [1]. In response to this challenge, we need to determine whether true reinfection occurs or if it is instead latent viral reactivation. There have been a few studies, including case reports and systematic literature reviews, on possible SARS-CoV-2 reinfection or viral relapse that contain the summarized clinical and epidemiological characteristics of these patients. This study includes one case report and evidence of review summarizing the clinical and epidemiological characteristics of patients with re-detectable positive SARS-CoV-2 virus. The aim of the present study was to explore whether the cases of suspected recurrence reported in the literature and in our clinic could be caused by a flawed sample or an incomplete viral clearance, or if effectively recovered people may be infected with a new strain of SARS-CoV-2. We also investigated whether reactivated COVID-19 can be contagious.

## Methods

### Case history

Patient A, a male student, 9 years old, was admitted to our hospital on September 28, 2020, due to having COVID-19 NAT^+^. He had tested NAT^−^ in Malawi on September 18 and 25. On September 27, 2020, he took a chartered flight from Malawi at 6:30 p.m. (China Standard Time) and arrived in Haikou, China, the next day at 1:40 p.m. He tested NAT^+^ on admission to China, the Ct value was 25, but he had no clinical symptoms such as nasal obstruction and mucus, cough, shortness of breath, cyanosis of the lips, nausea, vomiting, or diarrhea. His appetite and mental reactions were good. He took an ambulance to Hainan Provincial Hospital for isolation and treatment. His birth had been natural and breastfed, he grew up well, and he received his vaccinations on time. Physical examination on admission: body temperature, 36.5 °C; heart rate (HR), 110 beats per min (bpm); respiration rate (RR), 20×/min; weight, 60 kg; height, cm; and BMI, 26.67 (IOTF obesity cut point 22.77). Other physical examinations were normal.

Laboratory examination: white blood cell (WBC) count, 9.43 × 109 L; lymphocytes, 0.319; neutrophils, 0.584/µL; hemoglobin (Hb), 138 g/L; platelets, 397 × 10^9^/L; C-reactive protein (CRP), 1.26 mg/L; procalcitonin (PCT), 0.116 ng/mL. Liver functions: alanine transaminase (ALT), 268 U/L; aspartate transaminase (AST), 314 U/L; Erythrocyte sedimentation rate (ESR), renal function, myocardial-enzyme spectrum, electrolytes, immune function, ferritin, bleeding, and coagulation function were normal.

Pathogenic detection: Patient A was SARS-CoV-2 total aB+ (chemiluminescence), IgM−, and IgG+ (colloidal-gold method). Several SARS-CoV-2 NATs using nasal and anal swabs were negative. The patient was also negative for influenza A and B antigens, cytomegalovirus (CMV), and Epstein–Barr (EB) virus. Four items of respiratory etiology were normal. Antibodies for hepatitis viruses A, D, and E were negative. Chest computed tomography (CT) imaging showed no abnormalities other than the fatty liver.

**Fig. 1:**
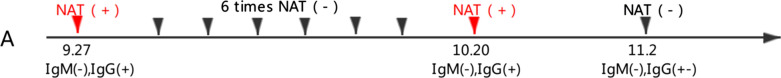
Timeline of Patient A

Patient A was admitted to the negative-pressure isolation ward of the Infectious Diseases (ID) department on September 28. Throat swabs, taken the same day, were negative for COVID-19 nucleic acids. He was treated with recombinant human interferon α2b spray, plus liver protection and symptomatic treatment using a compound glycyrrhizin injection and an ursodeoxycholic acid capsule. After testing negative on six NATs during the isolation period, he was transferred and quarantined at the hotel on October 12, where he was made comfortable. On October 20, his NAT throat swab was positive again, the Ct value was 31 (22 days between his first and last positive tests). He still had no fever, nasal congestion, runny nose, cough, cyanosis, wheezing, vomiting, diarrhea, skin rash, convulsion, or other COVID 19 symptoms. He was readmitted to the isolation ward, but his laboratory and imaging examinations were normal. In addition, he tested negative for SARS-CoV-2 NATs the next day and the days after. His ALT was 221 U/L, and his AST was 96 U/L in the follow-up tests on October 25 (Fig. 1).

### A: patient A

#### Epidemiological history

A stated, “Since the end of February 2020, my family had stayed at home in Malawi without going out, and the self-owned store was only managed by my husband.” A’s husband was diagnosed with COVID-19 infection locally on July 9, 2020, and he was the first confirmed case in this family. A’s husband was treated at home and self-quarantined after being confirmed. On August 5, A’s result was positive on July 16. A was self-quarantined at home for 20 days and took Chinese medicine for self-treatment. He was tested for SARS-CoV-2 nucleic acid at the agency company contracted with the Chinese embassy in Malawi on September 22 and 26, and the results were negative. Thus, she was declared healthy before boarding a plane. On September 27, A’s family of four (A, A’s husband, A’s mother, and A’s twin sister) took the direct international flight HU480 from Lilongwe, Malawi to Haikou, China, at 18:30 pm (Malawi time). They wore face masks throughout the journey and did not eat on the plane. A’s seat was 45 K. The flight arrived at Haikou Meilan International Airport at 13:40 pm on September 28 (Beijing time). After entering China, nasopharyngeal swabs and blood samples for SARS-CoV-2 nucleic acid, IgM, and IgG tests were collected from all passengers by Haikou Customs. Meanwhile, all passengers were transferred to a designated hotel for quarantine and observation. At 3:43 am, on September 29, the test results of A were reported positive for SARS-CoV-2 nucleic acid, total antibodies (chemiluminescence method), and IgG (colloidal gold method), but negative for IgM. On the morning of September 29, the local CDC collected throat swab specimens from A again for a SARS-CoV-2 nucleic acid retest, and the results were negative.

#### Next-generation sequencing (NGS)

Viral RNA was provided by the Centre for Disease Control of Hainan Province. We used a Qubit RNA HS kit (Thermo Fisher, Waltham, MA, USA; No. Q32855) to detect RNA concentrations in samples. Next, we performed polymerase chain reaction (PCR) amplification using the novel ultra-sensitive coronavirus genome-wide capture amplification kit from Micorfuture Company. PCR was performed with 30-min polymerase activation at 50 °C and then 2-min polymerase activation at 94 °C, followed by 40 cycles of denaturation at 94 °C for 15 s and annealing/polymerization at 65 °C for 5 min.

Next, we purified the PCR products using VAHTS Deoxyribonucleic Acid (DNA) Clean Beads (Vazyme Biotech Co., Ltd., Nanjing, China; No. N411). A DNA library was set up using a NEXTflex Rapid DNA-Seq Kit [Bioo Scientific (Perkin Elmer, Waltham, MA, USA); No. 5144-02] and a Dual DNA Adapter 96 Kit for Illumina (ABclonal Technology, Woburn, MA, USA; No. 20287). We performed PCR with 2-min polymerase activation at 98 °C, followed by 10 cycles of 98 °C for 30 s and 65 °C for 30 s. Polymerase extension was performed for 1 min at 72 °C. Next, we purified the library using VATHS DNA Clean Beads and then further diluted and denatured it with serine/threonine/tyrosine-protein kinase (HT1) and NaOH at a dilution concentration of 15 pM. We performed high-throughput sequencing (HTS) on an Illumina MiSeq (Illumina Inc., San Diego, CA, USA).

Sequencing reads were analyzed using the nCoV tool from Oxford Nanopore Technologies Ltd. (Oxford, UK). We first aligned the reads to the genome using Bowtie2 version 2.4.2 (http://bowtie-bio.sourceforge.net/bowtie2/, Oct 5, 2020) (Maryland, USA) and constructed the consensus sequence using local scripts. The parameters we used were minimum sequencing depth ≥ 1 and vote = 0.3.

#### Literature review

We searched the English-language literature in the PubMed, SpringerLink, and Elsevier databases and Chinese literature in the Wanfang and CNKI Chinese databases.

## Results

In total, we sequenced 10,726,434 paired-end reads for this patient. Approximately 80% of reads were aligned to the hCoV-19/Wuhan/IVDC-HB-01/2019 genome (EPI_ISL_402119). Based on the nCoV tool and local scripts, we obtained the consensus sequence, which was 17,235 bp in length. In exact terms, we first aligned the reads to the reference genome by Bowtie 2.4.2. Samtools software was used to sort and index the reads. For each base in the reference genome, we calculated the number of covered reads, the proportion of each base, and the proportion of insertion and deletion. If the proportion of the base was greater than 0.3, it suggested that this position was that base. Moreover, while this position had ≥ 2 bases with the proportion greater than 0.3, a heterozygous degenerate base was set in the consensus sequence. These results demonstrated that this patient was positive for SARS-CoV-2. Next, we compared this sequence with 702 published SARS-CoV-2 sequences and found it to be similar to the sequence from Kenya/P2601/2020.

A total of 168 articles were retrieved from PubMed using the terms “COVID-19, SARS-CoV-2, prolonged viral RNA presence/recurrence of positive/reactivation.” A total of 13 related reports were retrieved using the terms “COVID-19, SARS-CoV-2, prolonged viral RNA presence and child/recurrence of positive and child/reactivation and child.” Two related reports were retrieved using the terms “novel coronavirus, Fu Yang, infants, or children” from Chinese databases, but they were both reports of children with positive aBs and anal swabs after negative conversion of pharyngeal swabs and with positive nucleic acids on pharyngeal swabs once again after negative conversion of non-pharyngeal swabs. A total of 18 related reports were retrieved using the terms “novel coronavirus, Fu Yang.” After identifying valid cases of recurrent infection and combining them with our case, we found a total of 11 reports of SARS-CoV-2 reactivation, including 45 children (age range, newborn to 18 years). Sixteen males and nine females (with 20 Korean cases of unknown sex) had mild pneumonia or asymptomatic infection, and the interval between the first and last positive NATs was ≤ 50 days (minimum, 13 days). However, in the prior cases, there were no positive Ct values or SGS results. SGS results are reported in this case report, including HCOV-19/Wuhan/IVDC-HB-01/2019genome (EPI) strain 0 and Kenya/P2601/2020 strain 1. The results are summarized in Table 1 [2–12].

**Table 1:** Characteristics of patients

Refer- ences	Country (city)	Population size	Number of redetectable patients, n (child)	Sex (%/n)	Age (years)	Sample type	Interval between first and last NAT^+^ tests (days)	Symptoms during first episode	Symptoms during second episode	Treatment	Sample during reactivation as shown by NAT
Cases in article	China (Hainan)	1	1	M	9	Throat swabs, NAT	22	Symptomless	Symptomless	Symptomatic therapy	IgM^−^, IgG^+^; throat swabs, NAT
2	China (Wuhan)	–	1	M	8	Throat swabs, NAT	27	Fever; CT suggests lower lobe of left lung	Fever, chest CT improvement	Antiviral, symptomatic therapy	IgM^+/−^, IgG^+^; throat swabs, NAT
3	Korea	–	1	M	8	Throat swabs, qRT-PCR, anal swab	31	Cough; CT suggests lower lobe of left lung	Cough, low fever, CT chest−	Symptomatic therapy, nursing care	Throat swabs, NAT, anal swab
4	China (Shenzhen)	–	1	M	8	Throat swabs, anal swab, RT-PCR	50	Symptomless	Symptomless	Quarantine	Throat swabs, NAT
5	China (Beijing)	14	7	42.8%/ 3 M	2–7	Throat swabs, NAT	7–17 days after discharge	Two cases, asymptomatic; five cases, symptoms of cough, mild fever	Symptomless	3 cases, antiviral; 4 cases, symptomatic treatment	Throat swabs, NAT
6	China (Chongqing)	17	1	F	12	Throat swabs, anal swab, RT-PCR	34	Symptomless; CT^−^	Symptomless	Antiviral, symptomatic	Anal swab, RT-PCR
7	Korea	8922	20	–	0–18	Throat swabs, NAT	1–35 days after discharge	–	Symptomless or mild symptoms	–	Throat swabs, NAT
8	China (Guangzhou)	147	3	33.3%/ 1 M	4–16	Throat swabs, RT-PCR	22–32	Two cases, general; one case, mild	Symptomless	–	Throat swabs, RT-PCR; IgM^+/−^, IgG^+^
9	China (Beijing)	133	4	75%/ 3 M	2–10	Throat swabs, anal swab, RT-PCR	13–39	Symptomless or mild symptoms	Symptomless	–	Anal swab, RT-PCR
10	China (Dongguan)	7	3	66.6%/ 2 M	10 months to 14 years	Throat swabs, NAT	17–21	Symptomless or mild symptoms	Symptomless	Antiviral, symptomatic therapy	Anal swab, RT-PCR
11	China (Tianjin)	–	3	100%/ 3 M	6–9	Throat swabs, RT-PCR	17–27	Mild pneumonia, fever, nasal congestion, two mild GI tract symptoms	Symptomless	Antiviral, symptomatic therapy	Anal swab, RT-PCR
12	China (Hainan)	–	1	F	3 M	Throat swabs, RT-PCR	14	Fever, cough	Symptomless	Antiviral, symptomatic therapy	Anal swab, RT-PCR

*NAT* nucleic-acid test; *RT-PCR* reverse-transcription polymerase chain reaction; *IgG * immunoglobulin G; *IgM* immunoglobulin M

### Discussion

According to the records for Patient A, his throat swab was positive for SARS-CoV-2, but he had no fever, cough, nasal congestion, vomiting, or diarrhea, and his chest CT was normal. In such a case, a diagnosis of asymptomatic COVID-19 infection should be considered. However, the patient was COVID-19 IgM− and/or weakly COVID-19 IgG+ three consecutive times, with an interval of 22 days between the first and last positive tests. This leads one to wonder more than why, but also what the epidemiological implications might be. During this 22-day interval, how infectious was he? Is it caused by a flawed sample or an incomplete viral clearance, or can effectively recovered people be infected with a new strain of SARS-CoV-2? Can reactivated COVID-19 be contagious? Are appropriate disease control methods, such as strict isolation, needed with asymptomatic patients? These questions are worth answering.

Since December 2019, many cases of COVID-19 have been reported in Wuhan, Hubei Province [13]. Case reports on children have increased as the epidemic reached its peak. To date, the COVID-19 pandemic continues, and the number of confirmed cases worldwide is increasing. Researchers have found new variants of SARS-CoV-2, which reportedly can be detected in the respiratory and digestive tracts of the human body for long periods of time. In some COVID-19 patients, despite significant improvement in clinical symptoms, PCR has detected persistent or intermittent SARS-CoV-2. On February 25, the Guangdong Provincial Center for Disease Control and Prevention said at a press conference on the epidemic in Guangdong Province that 14% of COVID-19 patients discharged from provincial hospitals demonstrated the phenomenon of reactivation [14]. In a study of 70 COVID-19 patients, 21% of clinically recovered patients tested positive again after two consecutive negative NATs, with the longest duration of viral RNA positivity in this cohort being 45 days [15].

Li Tai Sheng et al.’s study of 37 adults with COVID-19 discharged from Wuhan hospitals confirmed the possibility that the virus can persist in patients. The average age of these patients was 62 years, and 64.9% were male. The majority of patients with SARS-CoV-2 infection showed remission of clinical symptoms, but long-term viral RNA in their bodies was still detectable. Of these patients, 78.4% denied any symptoms. A total of 431 PCR tests were performed (on average, eight per patient). By April 18, the median duration for PCR positivity was 78 days (interquartile range [IQR] 67.7–84.5), and the longest duration was 120 days. However, this does not necessarily mean that these patients were chronically infectious; the study found no secondary infections due to close contact with unprotected relatives. Of the 37 patients, 22 were discharged an average of 44 days after onset (IQR 22.3–50). Nine people lived with their families without personal protection, and no secondary infections were found through epidemiological investigations that included nucleic acid and aB screening. It has been suggested that the infectivity of patients with longstanding COVID-19 should not be evaluated only by reverse-transcription PCR (RT-PCR). The infectivity of those recovering clinically with a course of > 4 weeks is very limited [1]. Current disease prevention measures for patients with longstanding SARS-CoV-2 RNA might need to be reevaluated, although some researchers believe that the positive results could be due to incorrect sampling and/or testing methods [15]. For example, we found a positive anal-swab disjunction in a 3-month-old with COVID-19. Anal swabs were positive on each day of February 3–6, 2019 and negative on February 7 and 9, but positive again on February 13 and each day from February 17–19. We sent the anal-swab samples to another lab for a different test and found that they were still positive, with a Ct value of 30.04, suggesting the importance of proper sampling and/or confirmation with a different test [12].

To date, reported cases of SARS-CoV-2 reactivation shown by NATs are all in adults. This case report reviewed the clinical manifestations, epidemiological data, laboratory and imaging studies (especially NATs and SGS), treatment, and follow-up of a 9-year-old child with asymptomatic COVID-19 infection. We found that the Ct value of Patient A’s sample was 25/31/29, based on the positive nucleic acid Ct value provided by Chinese customs on September 28. respectively. Only when the CT value of within 33 can be NAT by the National Health Committee of the People’s Republic of China, the viral load was low, making it difficult to capture the metagenomic sequence. We sequenced genes from Patients A and B and found that only a few reads could be compared with the viral genome. On October 21, we once again used PCR to enrich Patient A’s sample and obtained enough viral nucleic acid to sequence it again.

In total, we sequenced 10,726,434 paired-end reads for this patient. Approximately 80% of reads were aligned to the hCoV-19/Wuhan/IVDC-HB-01/2019 genome (EPI_ISL_402119). Based on the nCoV tool and Artic pipeline, we obtained a 17,235 bp long consensus sequence. These results demonstrated that Patient A was COVID-19^+^. Next, we compared this sequence with 702 published COVID-19 sequences and found that it was most similar to the sequence from Kenya/P2601/2020. Viral tests of staff and materials at the hotel where Patient A was quarantined were negative. Patient A had three positive IgM tests and one positive total-aB test, which suggested that he had produced aBs protective against SARS-CoV-2, presumably due to having been infected in Malawi. Moreover, we found that the Ct value of Patient A’s positive NAT was very low, with no viral RNA captured by gene sequencing. This might have been due to the low content of the virus itself. We later obtained enough viral nucleic acid to sequence it. Patient A was infected in Malawi and was asymptomatic with protective aBs; the virus remained in his throat for a long time, but his viral load was low. In a prospective multicenter study, viruses were isolated from nasal swabs taken from 387 clinically recovered patients who showed low viral loads (quantification cycle value [Cq] > 30 over a quantifiable period). Median Cq was 36.8 (range 30.0–39.4). Cytopathic effect was detected in nine samples, and the corresponding positive culture rate was 2.3% (9/387) [16]. This shows that during the recovery period, the viral load of nucleic acid–positive patients decreases significantly. The virus replicates at a very low level, with no complete viral particles or residual fragments.

Generally speaking, after a viral pathogen invades the human body, there are three outcomes: pathogen elimination, a chronic pathogen-carrying state, or latent infection. Theoretically speaking, as long as viral nucleic acids can be detected, the patient is infectious. However, in terms of respiratory infectious diseases, asymptomatic infected and convalescent patients have no clinical symptoms such as cough and sputum, and the amount of virus they discharge is greatly reduced, so they should also be significantly less contagious. Thus, we suggest that adopting the concept of prolonged viral RNA presence instead of the concept of viral reactivation would provide us with a more accurate representation of such patients. If RT-PCR^+^ test results recur, then rapid retesting within a short period of time will help eliminate the technology caused by misjudgment; if necessary, DNA sequencing should be considered. Some experts believe that people who test positive for SARS-CoV-2 nucleic acids are infectious; the appearance of asymptomatic infected people might reinforce this belief, especially those in the recovery period. At present, there is no evidence that the people who were at the end of their quarantine periods with reactivated infections and no symptoms can infect others. An Italian study used third-generation gene sequencing and bioinformatics to analyze five samples, and reconstruction of the RNA sequence showed that the samples had only a few and very short gene fragments (< 600 nt). This finding suggests that residual RNA found by molecular detection does not function and represents only a fragment of the viral genome, which is degraded [16]. Patient A tested SARS CoV-2 IgM^−^ three times, IgG^+^ one time, and COVID-19 aB^+^ one time. Among the nucleic-acid-positive patients who were positive for SARS CoV-2 IgG, the time of onset of fever and weakness was 8–47 days, and the median number of days was 24. Based on the present median incubation period of SARS-CoV-2 infection, the median infection time of these patients was about 31 days, and they might have been SARS-CoV-2 IgM^−^. The combined detection of SARS-CoV-2 IgM and IgG is of great value in improving the clinical sensitivity of early COVID-19 detection. In terms of treatment monitoring and disease course, the decrease in or even disappearance of SARS-CoV-2 IgM concentration and the increase in SARS-CoV-2 IgG concentration indicate that patients gradually recover and develop immunity to the pathogenicity of COVID-19. Recently, it has been found that severity and prognosis of COVID-19 are affected by many factors, and anti-HCoV-OC43s IgG aB level is positively correlated with COVID-19 severity [17]. It is suggested that aB testing and third-generation gene sequencing should be carried out in patients with reactivated disease in order to assess viral load. If possible, specimens should be collected from multiple parts of the body, including sputum and stool samples, and epidemiological investigations of patients who test positive should continue to monitor their health and assess their infectiousness [18].

However, SARS-CoV-2 viral reactivation cannot be ruled out. Reactivation may have two possible mechanisms: (1) Prolonged infection. As part of the normal life cycle, SARS-CoV-2 viral RNA does not integrate into the host genome. It can persist in the respiratory tract and enter host cells. The viral genomic RNA is translated to produce nonstructural and structural proteins in the endoplasmic reticulum (ER) and presented on its surface to finish viron assembly and viral replication. The viral protein and replications triggered host protective immunity, which inhibits SARS-CoV-2 viral RNA replication, and viral RNA copies decrease to a very low level, which is undetectable using the current PCR NAT test, and patients seem to recover well. However, nature infection or asymptomatic COVID-19 infection is not sufficient to build up a sufficient immunity response or long-term protective immunity to clear out all SARS-CoV-2 virus in vivo. A slow viral clearance and prolonged carriage of the virus may predispose the patients to have a viral reactivation and displays re-detectable SARS-CoV-2 virus. (2) Latent viral reactivation. We hypothesize that as part of an abnormal life cycle, upon respiratory tract cell entry, SARS-CoV-2 genomic RNA could be hijacked by retroviral-like transposable elements and become integrated into host genome. After the initial infection, the host builds a strong immune response against SARS-CoV-2 and clears out all of the living virus, and patients are not re-exposed to the virus. However, the integrated virus could still survive in the host genome in the absence of evidence for viral replication. SARS-CoV-2 could be periodically reactivated and give rise to the expression of viral sequences detectable by PCR when its host immunity fades away. Taking all this into consideration, at present, there is no evidence that people who are at the end of their quarantine periods with reactivated infections and no symptoms can infect others.

### Limitations

A child’s memory bias is large, and diagnosis of asymptomatic infection was mainly based on clinical and laboratory results.
